# Correction: Spatial frequency adaptation modulates population receptive field sizes

**DOI:** 10.7554/eLife.109758

**Published:** 2025-10-31

**Authors:** Ecem Altan, Catherine A Morgan, Steven C Dakin, D Samuel Schwarzkopf

**Keywords:** Human

Altan E, Morgan CA, Dakin SC, Schwarzkopf DS. 2025. Spatial frequency adaptation modulates population receptive field sizes. *eLife* **13**:RP100734. doi: 10.7554/eLife.100734.

Published 26 September 2025

The description of how we calculated the noise ceiling of the fMRI data under Methods subsection “Preprocessing” is incorrect. However, the formula used in the analysis is correct and the results are unchanged.

For clarity, the noise ceiling of the goodness of fit is identical to the output of the Spearman-Brown prophecy formula, $\rho^{\ast} _{xx'}$, which predicts the test-retest correlation of a full dataset (Spearman, 1910; Brown, 1910):

$$\displaystyle \rho^{\ast} _{xx'}=\frac{2\rho _{xx'}}{1+\rho _{xx'}}$$

where $\rho _{xx'}$ is the test-retest correlation of the split half data.

The descriptive error in the original version of this article arose by conflation of text within two previous articles we cited for this procedure (Morgan & Schwarzkopf, 2020; Urale et al., 2022). Both procedures result in the same outcome but arrive there through a slightly different route.

Corrected text: “*The noise ceiling is equal to this reliability measure as it denotes a maximum achievable goodness of fit for each vertex*.”

Original text for reference: *“The noise ceiling is achieved by squaring this measure, which gives a maximum achievable goodness of fit for each vertex.”*

The article has been corrected accordingly. We apologise for any confusion this may have caused.

